# The Functional Head Impulse Test to Assess Oscillopsia in Bilateral Vestibulopathy

**DOI:** 10.3389/fneur.2019.00365

**Published:** 2019-04-16

**Authors:** T. S. van Dooren, F. M. P. Lucieer, S. Duijn, A. M. L. Janssen, N. Guinand, A. Pérez Fornos, V. Van Rompaey, H. Kingma, S. Ramat, R. van de Berg

**Affiliations:** ^1^Division of Balance Disorders, Department of Otorhinolaryngology and Head and Neck Surgery, Maastricht University Medical Centre, Maastricht, Netherlands; ^2^Faculty of Health, Medicine and Life Sciences, University of Maastricht, Maastricht, Netherlands; ^3^Department of ENT/Audiology, School for Mental Health and Neuroscience (MHENS), Maastricht University Medical Centre, Maastricht, Netherlands; ^4^Department of Methodology and Statistics, Care and Public Health Research Institute (CAPHRI), Maastricht University, Maastricht, Netherlands; ^5^Service of Otorhinolaryngology Head and Neck Surgery, Department of Clinical Neurosciences, Geneva University Hospitals, Geneva, Switzerland; ^6^Faculty of Medicine and Health Sciences, University of Antwerp, Antwerp, Belgium; ^7^Department of Otorhinolaryngology and Head and Neck Surgery, Antwerp University Hospital, Edegem, Belgium; ^8^Faculty of Physics, Tomsk State Research University, Tomsk, Russia; ^9^Department of Computer, Electric and Biomedical Engineering, University of Pavia, Pavia, Italy

**Keywords:** functional head impulse test (fHIT), dynamic visual acuity (DVA), Oscillopsia, oscillopsia severity questionnaire, functional vestibular testing, bilateral vestibulopathy (BV)

## Abstract

**Introduction:** Bilateral vestibulopathy (BV) is a chronic condition in which vestibular function is severely impaired or absent on both ears. Oscillopsia is one of the main symptoms of BV. Oscillopsia can be quantified objectively by functional vestibular tests, and subjectively by questionnaires. Recently, a new technique for testing functionally effective gaze stabilization was developed: the functional Head Impulse Test (fHIT). This study compared the fHIT with the Dynamic Visual Acuity assessed on a treadmill (DVA_treadmill_) and Oscillopsia Severity Questionnaire (OSQ) in the context of objectifying the experience of oscillopsia in patients with BV.

**Methods:** Inclusion criteria comprised: (1) summated slow phase velocity of nystagmus of <20°/s during bithermal caloric tests, (2) torsion swing tests gain of <30% and/or phase <168°, and (3) complaints of oscillopsia and/or imbalance. During the fHIT (Beon Solutions srl, Italy) patients were seated in front of a computer screen. During a passive horizontal head impulse a Landolt C optotype was shortly displayed. Patients reported the seen optotype by pressing the corresponding button on a keyboard. The percentage correct answers was registered for leftwards and rightwards head impulses separately. During DVA_treadmill_ patients were positioned on a treadmill in front of a computer screen that showed Sloan optotypes. Patients were tested in static condition and in dynamic conditions (while walking on the treadmill at 2, 4, and 6 km/h). The decline in LogMAR between static and dynamic conditions was registered for each speed. Every patient completed the Oscillopsia Severity Questionnaire (OSQ).

**Results:** In total 23 patients were included. This study showed a moderate correlation between OSQ outcomes and the fHIT [rightwards head rotations (*r*_s_ = −0.559; *p* = 0.006) leftwards head rotations (*r*_s_ = −0.396; *p* = 0.061)]. No correlation was found between OSQ outcomes and DVA_treadmill_, or between DVA_treadmill_ and fHIT. All patients completed the fHIT, 52% of the patients completed the DVA_treadmill_ on all speeds.

**Conclusion:** The fHIT seems to be a feasible test to quantify oscillopsia in BV since, unlike DVA_treadmill_, it correlates with the experienced oscillopsia measured by the OSQ, and more BV patients are able to complete the fHIT than DVA_treadmill_.

## Introduction

Gaze stabilization is one of the many functions of the vestibular system. The vestibulo-ocular reflex (VOR) enables gaze stabilization during high-frequency head movements by moving the eyes directly in opposite direction of the head movement. A decreased VOR therefore impairs gaze stabilization, which leads to head or body movement-induced blurred vision (oscillopsia). Oscillopsia is one of the main symptoms of bilateral vestibulopathy (BV) (1).

BV is a heterogeneous chronic condition in which vestibular function is severely impaired or absent on both ears (2). BV patients have a variety of symptoms and report a significant reduction in quality of life. Therapeutic options are often limited to balance training, but studies are now focusing on restoring vestibular function with a vestibular implant (3–6).

To treat patients with BV, the condition must be first recognized by clinicians. The diagnosis of BV is often under- or misdiagnosed. Therefore, sufficient inclusion criteria and validated patient-reported outcome measures are needed for patients with BV. One of the components is to quantify the experience of oscillopsia in BV patients (2, 7).

Oscillopsia can be quantified subjectively by questionnaires, such as the Oscillopsia Severity Questionnaire (OSQ) (8). These questionnaires are designed to classify the disease burden experienced by patients in daily life. Additionally, oscillopsia can be quantified objectively by functional vestibular tests that assess dynamic visual acuity (DVA) (9, 10). Various clinical testing paradigms have been proposed to assess DVA, like walking on a treadmill or passively shaking the head, while reading an optotype chart (8, 11). A new technique was recently suggested: the functional head impulse test (fHIT). The fHIT provides information about the functional performance of the rotational VOR by testing its gaze stabilization ability during passive head impulses in a range of peak head accelerations from 3,000 to 6,000 deg/s^2^ (12–15).

The aim of this study was to compare the fHIT with the DVA on a treadmill (DVA_treadmill_) and OSQ outcomes in the context of quantifying oscillopsia in BV patients. Preliminary data from our laboratory showed inter- and intrasubject discrepancies between fHIT and DVA_treadmill_ results in patients with BV. This might be the result of the different stimuli applied during these tests: fHIT selectively stimulates the horizontal semicircular canals with passive head movements, while DVA_treadmill_ stimulates the whole vestibular system with active whole-body movements. Based on these experiences, it was hypothesized that: (1) fHIT and DVA_treadmill_ differ with respect to quantifying oscillopsia since different stimuli are given, and (2) therefore one of them might correlate better to the OSQ.

## Methods

### Study Population

This study comprised patients diagnosed with BV at the Division of Balance Disorders at Maastricht University Hospital. Inclusion criteria were: (1) summated slow phase velocity of nystagmus of <20°/s during biothermal caloric tests (30 and 44°C, 300 mL in 30 s), (2) torsion swing tests gain of <30% and/or phase <168° (peak velocity of 60°/s; sinusoidal rotation 0.11Hz), and (3) complaints of oscillopsia and/or imbalance. The inclusion criteria differed on some aspects from the diagnostic criteria of BV from the Báràny Society, since inclusion of this study started before these criteria were published (1). Based on normative data in our laboratory, the lower limit of a normal caloric test on one side is a sum of bithermal slow phase velocities of nystagmus of 25°/s (15°/s warm, 10°/s cold). BV patients included in this study had a maximum sum of bithermal slow phase velocities of nystagmus on one side of 15°/s. In this study, some patients will not perfectly fit the BV criteria from the Báràny Society, nonetheless they definitely have a bilateral vestibular dysfunction (see Supplementary Material).

Exclusion criteria comprised peripheral neuropathy, being unable to stop vestibular suppressants for one week (cinnarizine and all psychiatric medication), or the inability to walk independently.

### Testing

Every patient underwent fHIT and DVA_treadmill_ on one day in the same order, and with a break in between. Both tests were performed by one trained examiner (FL) under standardized conditions, in the same room with controlled illumination. Patients were tested binocularly and corrective spectacles or contact lenses were worn during fHIT, and removed during DVA_treadmill_.

#### Functional Head Impulse Test (fHIT) (12–14)

The fHIT was performed using the fHIT system (Beon Solutions srl, Zero Branco (TV), Italy). Patients were seated in a static chair in front of a computer screen at a distance of 1.5 meter with a keyboard in their hand. During a passive head impulse, when head acceleration reached its peak value, an optotype (Landolt C ring) was displayed on the screen for 80 ms. The size of the optotype was adjusted for every subject separately, and remained constant during testing. Before the start of the fHIT, the static visual acuity threshold was acquired by the fHIT system in 20 trials. Optotype size started from 1.0 LogMAR (log of the Minimum Angle of Resolution) and decreased depending on the subjects' rates of errors. The used optotype size was equal to this threshold, increased by 0.6 LogMAR (13). During fHIT, patients had to choose the right optotype out of eight different options by pressing the corresponding button on the keyboard. No direct feedback was given. Head impulses comprised fast (peak velocity >150°/s) (16, 17), outwards, passive, horizontal rotational head movements with a low amplitude (±20°), unpredictable in timing and direction. At least 10 impulses were given to both sides. The absolute outcome was the percentage of correct answers (%CA) for each side, as calculated by the fHIT system. A %CA of <80 was considered abnormal. This cut-off was a conservative approximation of the criterion adopted by the fHIT system, which considers the level where the standardized normal deviate of the patient falls outside the 99% of the two-tailed Z distribution of a population of age-matched controls (14).

#### DVA_treadmill_

DVA was assessed on a treadmill (1210 model, SportsArt, Inc., Tainan, Taiwan, China) with a computer screen placed at a distance of 2.8 meters from the subject. Sloan letter optotypes were used. Testing started with optotypes presented at a LogMAR of 1.0. When four out of five optotypes were recognized correctly, the corresponding LogMAR was considered achieved and the size was decreased by steps of 0.1 LogMAR. When three or less optotypes were recognized correctly, the corresponding LogMAR was considered unachieved. The best (i.e., lowest) achieved LogMAR was recorded. Patients were tested in static condition (while standing still) and in dynamic conditions (while walking on the treadmill at 2, 4, and 6 km/h). Every condition was tested once. In case the patient was not able to walk independently at a certain speed, the test was stopped and registered as impossible for that speed. Absolute outcome for every speed was the visual acuity difference (VA difference), calculated as the decline in LogMAR between static and dynamic conditions. DVA_treadmill_ was considered abnormal when a VA difference of >0.2 was recorded at 2 and 4 km/h or >0.3 at 6 km/h (8, 18, 19).

#### Oscillopsia Severity Questionnaire (OSQ)

Every patient completed the oscillopsia severity questionnaire (OSQ) developed by the Division of Balance Disorders in Maastricht. The OSQ consists of nine questions about the patients' experience of oscillopsia in daily life, as shown in Table 1. Every question can be answered by one of the following five options Always (= 5), Often (= 4), Sometimes (= 3), Seldom (= 2), or Never (= 1). The outcome of every separate question was registered and the mean value for every patient was calculated. A mean value of three or more was considered as moderate to extreme oscillopsia severity (8, 20).

**Table 1:** The oscillopsia severity questionnaire (OSQ).

**OSCILLOPSIA SEVERITY QUESTIONNAIRE**	
1.	Do you have the sensation that the visual environment is moving when it's not?
2.	By dim light, do you have the sensation that the visual environment is not stable?
3.	Is it difficult for you to recognize known faces when you are walking?
4.	When you are reading, do you have the sensation that the text is not stable?
5.	When you are watching television, do you have the sensation that the image is not stable?
6.	When you are driving your car, do you have the sensation that the visual environment is not stable?
7.	As a car passenger, do you have the sensation that the visual environment is not stable?
8.	When you are riding a bicycle, do you have the sensation that the visual environment is not stable?
9.	When you are walking on uneven ground, do you have the sensation that the visual environment is not stable?

*Questions can be answered with Always = 5, Often = 4, Sometimes = 3, Seldom = 2, or Never = 1. A mean value of 3 or more was considered as moderate to extreme oscillopsia severity*.

### Statistical Analysis

Data were analyzed using SPSS Statistics 24 for Windows. Significance was set on *p* < 0.05. Bonferroni correction was used in case of multiple comparisons. The Shapiro-Wilk test, and visual inspection of the histogram and normal Q-Q plot of the outcome distributions were used to determine whether the data were normally distributed. In case there was no normal distribution of data, non-parametric tests (Wilcoxon Sign-Rank test, McNemar, Mann-Whitney U or Spearman's Rank Correlation test) were used.

The correlation was calculated between fHIT and DVA_treadmill_, between DVA_treadmill_ (VA difference) at 2, 4, and 6 km/h and OSQ score, and between fHIT (%CA) and OSQ score. Duration of illness was compared between DVA_treadmill_ outcome and OSQ score, and between fHIT outcome and OSQ score.

During further analyses 3 groups were differentiated: (1) fHIT abnormal vs. normal for rightwards and leftwards head rotations. In case fHIT was abnormal to at least one side, the outcome was considered abnormal during this analysis. (2) DVA_treadmill_ impossible vs. possible. The impossible subgroup consists of patients that were not able to walk independently at 2, 4, and/or 6 km/h. (3) DVA_treadmill_ abnormal vs. normal. During this analysis patients with an impossible DVA_treadmill_ at any speed were considered missing data. Within these groups, OSQ outcomes were compared between the subgroups.

### Ethical Considerations

This study was in accordance with the Declaration of Helsinki (amended version 2013). Approval was obtained from the ethical committees of Maastricht University Medical Center (NL52768.068.15/METC151027). All participants provided written informed consent prior to the study.

## Results

In this study 23 patients with BV were included, 13 male and 10 female. Mean age was 57.6 (SD 11.04). Duration of illness varied between 18 months and 33 years. Etiologies comprised: ototoxicity due to gentamicin treatment (3) or chemotherapy (1), post-infectious due to Lyme disease (1) or meningitis (1), DFNA-9 gene mutation (3), bilateral Ménière's disease (2), autoimmune disease (1). In 10 patients, no cause could be found (idiopathic).

### fHIT

**Figure 1:**
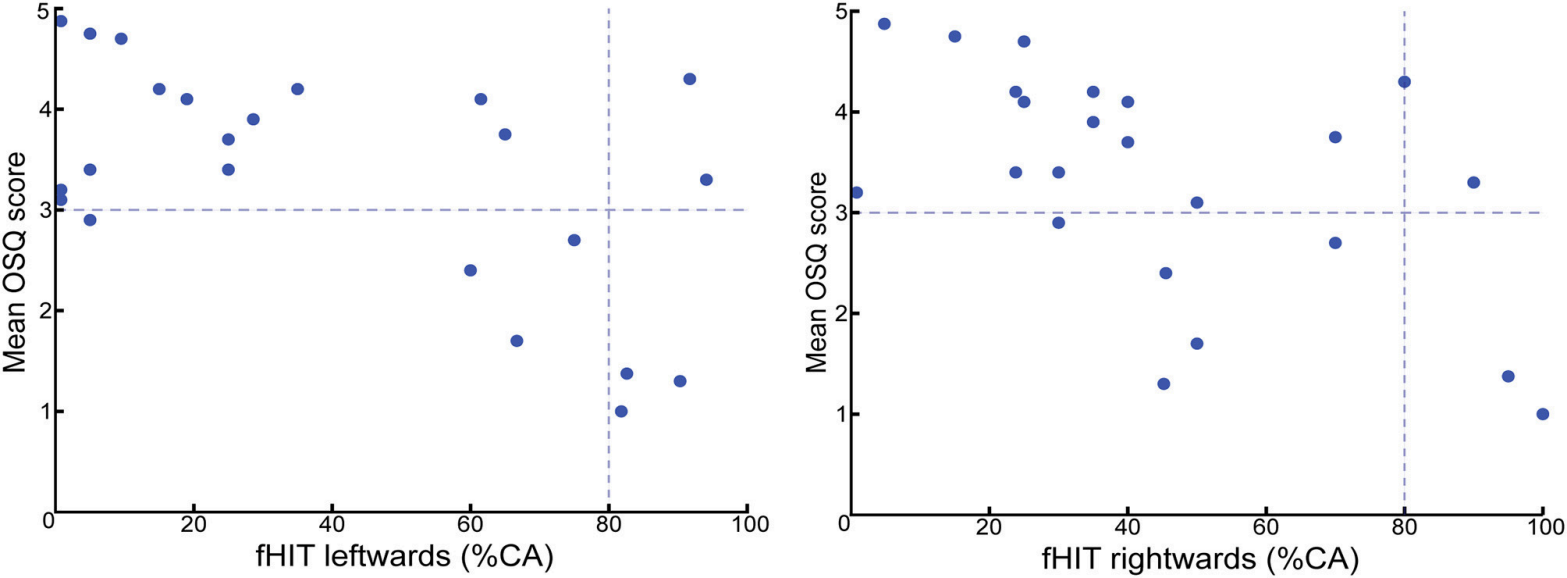
fHIT outcome (percentage of correct answers, %CA) vs. mean OSQ score. The horizontal interceptive line represents the cut-off value of the OSQ; a value of three or more is considered as moderate to extreme oscillopsia severity. The vertical interceptive line represents the cut-off value of the fHIT; a %CA-value of <80 was considered abnormal. This study showed a moderate correlation between the severity of oscillopsia tested by the OSQ, and percentage of correct answers on the fHIT for both rightwards (*r*_s_ = −0.559; *p* = 0.006) and leftwards (*r*_s_ = −0.396; *p* = 0.061) head impulses.

All 23 patients (100%) completed the fHIT. Outcomes for rightwards and leftwards head rotations did not significantly differ. Eighteen patients (78%) showed an abnormal fHIT to both sides, and four patients (17%) had normal fHIT outcomes. One patient (4%) had a unilateral abnormal fHIT: 45%CA on the right side and 90%CA on the left side. No significant difference was found in OSQ score between patients with a normal and abnormal fHIT. A moderate correlation was found between %CA on the fHIT and OSQ score for rightwards (*r*_s_ = −0.559, *p* = 0.006) and leftwards (*r*_s_ = −0.396, *p* = 0.061) head impulses (Figure 1).

### DVA_treadmill_

In total 12 BV patients (52%) completed the DVA on all three speeds. With increasing speed, the number of patients that could not walk independently (and not complete the test) increased: two patients at 2 km/h and 11 patients at 6 km/h. VA difference between 2, 4, and 6 km/h did not differ statistically significant. DVA, at any speed, was only abnormal in four patients (17%). All four patients showed abnormal DVA at 4 km/h, and one even at 2 km/h. Of these four patients, neither completed a walking speed of 6 km/h. (Table 2) Mean OSQ outcome and duration of illness did not differ significantly between patients with a normal or abnormal DVA or between patients with a possible or impossible DVA. No correlation was found between OSQ outcome and the amount of VA difference at any speed.

**Table 2:** DVA_treadmill_ outcomes.

	**DVA 2 km/h**	**DVA 4 km/h**	**DVA 6 km/h**	DVA all speeds^*^
Normal	20 (87%)	16 (70%)	12 (52%)	8 (35%)
Abnormal	1 (4%)	4 (17%)	0 (0%)	4 (17%)
Not possible	2 (9%)	3 (13%)	11 (48%)	11 (48%)

DVA_treadmill_ was considered abnormal when a VA difference of >0.2 was recorded at 2 and 4 km/h or >0.3 at 6 km/h. In case a patient could not walk a certain speed independently, this speed was classified as “not possible.”

* *DVA at all speeds was classified as “not possible” or “abnormal” when a patient did not complete the DVA_treadmill_ protocol at all speeds or had an abnormal outcome at one or more speeds*.

### fHIT vs. DVA_treadmill_

fHIT showed more abnormal outcomes than DVA_treadmill_ at all speeds: 78% vs. 17%. Next to this, fHIT was possible in all 23 patients, while DVA_treadmill_ could not be completed in 11 of them. All four patients with abnormal DVA_treadmill_ outcomes, showed abnormal bilateral fHIT outcomes as well. No correlation between fHIT and DVA_treadmill_ was found at any tested speed (2, 4, 6 km/h), for both rightwards and leftwards head rotations.

## Discussion

This study compared the fHIT with DVA assessed on a treadmill, and OSQ outcomes in the context of quantifying oscillopsia in patients with BV. fHIT outcomes showed a moderate correlation with the experienced oscillopsia in daily life, as assessed by the OSQ. DVA_treadmill_ outcomes, at any of the tested speeds, did not correlate to the severity of oscillopsia, as measured by OSQ. This is in agreement with previous studies with a large study population of BV patients (8). There is no gold standard for measuring oscillopsia, this study used the oscillopsia severity questionnaire (OSQ) to capture the subjective complaints of BV patients. (8) Specific questions from this questionnaire—those with highest correlation with fHIT—could possibly be of value in establishing a validated patient reported outcome measures for BV (7).

fHIT showed more abnormal outcomes than DVA_treadmill_ at all speeds (78 vs. 17%). This is probably due to multiple factors (9). First, the ability to compensate or adapt is less during fHIT than during DVA_treadmill_. During walking on a treadmill, patients are able to use compensation mechanisms to improve gait or gaze stabilization (e.g., by trying to minimize the overall head movement). Secondly, an active movement is made during DVA_treadmill_, in contrast to the passive movement during fHIT. Passive movements have been shown to be most useful in discriminating between healthy subjects and patients with bilateral vestibular loss (16, 21). Indeed, during walking an efference copy of the command producing the walking movement is available, thereby allowing patients to predict the retinal slippage as a consequence on the resulting head movement (22). Thirdly, the nature of the stimulus differs between the two tests. The fHIT selectively stimulates the plane of one semi-circular canal during passive head movements in high frequencies (>150°/s), while DVA_treadmill_ comprises an active movement which stimulates all semi-circular canals and otoliths at the same time (14). The frequency of the stimulus depends on the walking speed. When walking at a speed of 6 km/h, angular velocities are ~178°/s, and lateral and horizontal head translations occur at 1 Hz and 2 Hz, respectively (23).

BV criteria, and the inclusion criteria of this study, comprise low or absent function of the horizontal semi-circular canal. In case the patient had residual function of other sensory parts of the vestibular system (i.e., the otoliths), it could be possible that this residual function was used during DVA_treadmill_. This possible selection bias could lead to false negative DVA_treadmill_ outcomes. These mechanisms might also (partially) explain why the fHIT has a stronger correlation to oscillopsia experience than DVA_treadmill_.

Comparing the ability of subjects to complete a test, fHIT could be performed in more patients than DVA_treadmill_. After all, in this study population 100% of the patients was able to complete the fHIT, while 87% of the patients completed the DVA-protocol at 4 km/h and only 52% at 6 km/h. The inability to walk faster than 5 km/h on a treadmill in BV patients was described in previous studies (11, 24).

A possible limitation of this study is the fact that DVA_treadmill_ was tested without wearing any corrective spectacles. It is unlikely this has influenced the outcomes, since DVA_treadmill_ outcome (VA difference) was calculated as the decline in LogMAR in a patient between static and dynamic conditions, both tested without corrective spectacles. Furthermore, different DVA_treadmill_ cut-off values are reported in literature (1, 8, 18, 19). In this study, cut-off values were based on walking-speed-specific normative values from the vestibular laboratory of the Maastricht University Medical Center. Despite the fact this study showed a moderate correlation between fHIT and OSQ, the correlation between objective and subjective tests to quantify oscillopsia is not (yet) optimal. It is possible that the used questionnaire (OSQ) captures more complaints than only oscillopsia and can be influenced by a patients' coping of BV. Lastly, in this article fHIT and DVA_treadmill_ are compared. Both tests give different stimuli to the vestibular system, as described above, and are therefore never fully comparable.

To summarize, the fHIT seems feasible for quantifying oscillopsia in patients with BV. In the future, it possibly could also be used to measure functional outcome in patients implanted with a Vestibular Implant.

## Conclusion

The functional Head Impulse Test (fHIT) is a recently proposed technique to assess functionally effective gaze stabilization. The fHIT seems to be a feasible test to objectify oscillopsia in BV since, unlike DVA assessed on a treadmill, it correlates with the experienced oscillopsia measured by the OSQ, and more BV patients are able to complete the fHIT than DVA assessed on a treadmill.

## Ethics Statement

This study was in accordance with the Declaration of Helsinki (amended version 2013). Approval was obtained from the ethical committees of Maastricht University Medical Center (NL52768.068.15/METC151027). All participants provided written informed consent prior to the study.

## Author Contributions

HK, SR, and RvdB: design of the work; FL: acquisition; TvD, SD, and AJ: analysis; TvD, FL, NG, AP, VV, HK, SR, and RvdB: interpretation; TvD, FL, SD, AJ, NG, AP, VV, HK, SR, and RvdB: revising the work, final approval of the version to be published, and agreement to be accountable for all aspects of the work in ensuring that questions related to the accuracy or integrity of any part of the work are appropriately investigated and resolved.

### Conflict of Interest Statement

SR is the author of a Patent Deposit Application regarding the technique used in the functional head impulse test and is a shareholder of the company producing of the fHIT system used in this study [Beon Solutions srl, Zero Branco (TV), Italy]. The remaining authors declare that the research was conducted in the absence of any commercial or financial relationships that could be construed as a potential conflict of interest.

## References

[1] Strupp M Kim JS Murofushi T Straumann D Jen JC Rosengren SM. Bilateral vestibulopathy: diagnostic criteria consensus document of the classification committee of the Barany society. J Vestib Res. (2017) 27:177–89. 10.3233/VES-170619 29081426 PMC9249284

[2] Lucieer F Vonk P Guinand N Stokroos R Kingma H van de Berg R. Bilateral vestibular hypofunction: insights in etiologies, clinical subtypes, and diagnostics. Front Neurol. (2016) 7:26. 10.3389/fneur.2016.00026 26973594 PMC4777732

[3] van de Berg R Guinand N Ranieri M Cavuscens S Khoa Nguyen TA Guyot JP. The vestibular implant input interacts with residual natural function. Front Neurol. (2017) 8:644. 10.3389/fneur.2017.00644 29312107 PMC5735071

[4] van de Berg R Guinand N Nguyen TA Ranieri M Cavuscens S Guyot JP The vestibular implant: frequency-dependency of the electrically evoked vestibulo-ocular reflex in humans. Front Syst Neurosci. (2014) 8:255 10.3389/fnsys.2014.00255 25653601 PMC4299437

[5] van de Berg R Guinand N Stokroos RJ Guyot JP Kingma H. The vestibular implant: quo vadis? Front Neurol. (2011) 2:47. 10.3389/fneur.2011.00047 21991260 PMC3181464

[6] Guinand N Van de Berg R Cavuscens S Stokroos R Ranieri M Pelizzone M. Restoring visual acuity in dynamic conditions with a vestibular implant. Front Neurosci. (2016) 10:577. 10.3389/fnins.2016.00577 28066163 PMC5177740

[7] Lucieer F Duijn S Van Rompaey V Pérez A Guinand N Guyot JP. Full spectrum of reported symptoms of bilateral vestibulopathy needs further investigation-a systematic review. Front Neurol. (2018) 9:352. 10.3389/fneur.2018.00352 29915554 PMC5994412

[8] Guinand N Pijnenburg M Janssen M Kingma H. Visual acuity while walking and oscillopsia severity in healthy subjects and patients with unilateral and bilateral vestibular function loss. Arch Otolaryngol Head Neck Surg. (2012) 138:301–6. 10.1001/archoto.2012.4. 22431876

[9] van de Berg R van Tilburg M Kingma H. Bilateral vestibular hypofunction: challenges in establishing the diagnosis in adults. ORL J Otorhinolaryngol Relat Spec. (2015) 77:197–218. 10.1159/000433549 26366566

[10] Anson ER Gimmon Y Kiemel T Jeka JJ Carey JP. A tool to quantify the functional impact of oscillopsia. Front Neurol. (2018) 9:142. 10.3389/fneur.2018.00142 29599743 PMC5862789

[11] Herdman SJ Tusa RJ Blatt P Suzuki A Venuto PJ Roberts D. Computerized dynamic visual acuity test in the assessment of vestibular deficits. Am J Otol. (1998) 19:790–6. 9831156

[12] Bohler A Mandala M Ramat S. A software program for the head impulse testing device (HITD). Conf Proc IEEE Eng Med Biol Soc. (2010) 2010:6615–8. 10.1109/IEMBS.2010.5627138 21096520

[13] Colagiorgio P Colnaghi S Versino M Ramat S. A new tool for investigating the functional testing of the VOR. Front Neurol. (2013) 4:165. 10.3389/fneur.2013.00165 24298265 PMC3829465

[14] Ramat S Colnaghi S Boehler A Astore S Falco P Mandalà M. A device for the functional evaluation of the VOR in clinical settings. Front Neurol. (2012) 3:39. 10.3389/fneur.2012.00039 22470364 PMC3311056

[15] Corallo G Versino M Mandalà M Colnaghi S Ramat S. The functional head impulse test: preliminary data. J Neurol. (2018) 265 (Suppl. 1):35–9. 10.1007/s00415-018-8910-z 29868981

[16] Vital D Hegemann SC Straumann D Bergamin O Bockisch CJ Angehrn D. A new dynamic visual acuity test to assess peripheral vestibular function. Arch Otolaryngol Head Neck Surg. (2010) 136:686–91. 10.1001/archoto.2010.99 20644064

[17] MacDougall HG Weber KP McGarvie LA Halmagyi GM Curthoys IS. The video head impulse test: diagnostic accuracy in peripheral vestibulopathy. Neurology. (2009) 73:1134–41. 10.1212/WNL.0b013e3181bacf85 19805730 PMC2890997

[18] Fife TD Tusa RJ Furman JM Zee DS Frohman E Baloh RW. Assessment: vestibular testing techniques in adults and children: report of the therapeutics and technology assessment subcommittee of the American academy of neurology. Neurology. (2000) 55:1431–41. 10.1212/WNL.55.10.1431 11094095

[19] Hain TC Cherchi M Yacovino DA. Bilateral vestibular loss. Semin Neurol. (2013) 33:195–203. 10.1055/s-0033-1354597 24057822

[20] Guinand N Boselie F Guyot JP Kingma H. Quality of life of patients with bilateral vestibulopathy. Ann Otol Rhinol Laryngol. (2012) 121:471–7. 10.1177/000348941212100708 22844867

[21] Tian JR Shubayev I Demer JL. Dynamic visual acuity during passive and self-generated transient head rotation in normal and unilaterally vestibulopathic humans. Exp Brain Res. (2002) 142:486–95. 10.1007/s00221-001-0959-7 11845244

[22] Bhansali SA Stockwell CW Bojrab DI. Oscillopsia in patients with loss of vestibular function. Otolaryngol Head Neck Surg. (1993) 109:120–5. 8336958 10.1177/019459989310900122

[23] Moore ST Hirasaki E Raphan T Cohen B. The human vestibulo-ocular reflex during linear locomotion. Ann N Y Acad Sci. (2001) 942:139–47. 11710456 10.1111/j.1749-6632.2001.tb03741.x

[24] Lambert S Sigrist A Delaspre O Pelizzone M Guyot JP. Measurement of dynamic visual acuity in patients with vestibular areflexia. Acta Otolaryngol. (2010) 130:820–3. 10.3109/00016480903426592 20082568

