# Supplementary material for: The Functional Head Impulse Test to Assess Oscillopsia in Bilateral Vestibulopathy
Source: Front Neurol. 2019 Apr 16;10:365. doi: 10.3389/fneur.2019.00365 (PMC6499172; doi:10.3389/fneur.2019.00365)
Supplement: Supplementary file 1 [file Table_1.pdf]

				Calorics (slow phase velocity of nystagmus in °/s)					Torsion swing		vHIT	
	**Etiology**	**Age**	**Sex**	**Warm right**	**Warm left**	**Cold left**	**Cold right**	**Sum**	**Gain** (%)	**Phase** (°)	**VOR-gain left**	**VOR-gain right**
**1**	Idiopathic	53	M	0	0	0	0	0	1	-132	0,07	0,03
**2**	Gentamycin	74	V	6	0	0	0	6	41	159	0,46	0,48
**3**	Idiopathic	47	M	7	0	0	7	14	22	158	0,28	0,62
**4**	Gentamycin	79	M	0	0	0	0	0	2	28	0,46	0,35
**5**	Idiopathic	49	M	0	0	0	0	0	3	-159	0,08	0,02
**6**	Gentamycin	60	M	0	0	0	0	0	7	-197	0,56	0,44
**7**	DFNA-9	58	M	0	0	0	0	0	4	129	0,17	0,03
**8**	Ménière's disease ADS	69	V	2	3	5	2	12	52	143	0,88	0,92
**9**	Idiopathic	36	M	8	0	0	7	15	18	-172	0,49	0,72
**10**	Lyme disease	42	M	7	1	0	2	10	7	-228	0,57	0,58
**11**	DFNA-9	62	M	0	0	0	0	0	2	24	0,26	0,17
**12**	Idiopathic	55	V	0	0	0	0	0	0	302	0,17	0,11
**13**	Idiopathic	49	V	0	0	0	0	0	10	-246	0,32	0,39
**14**	Auto-immune	65	M	0	0	0	0	0	5	209	0,39	0,47
**15**	Idiopathic	67	V	0	0	0	0	0	34	-222	0,71	0,31
**16**	Bacterial meningitis	55	V	0	10	3	0	13	12	146	0,34	0,29
**17**	Ménière's disease ADS	75	V	9	0	0	0	9	8	129	0,83	0,89
**18**	Idiopathic	65	M	0	2	0	5	7	26	-242	0,33	0,54
**19**	Idiopathic	51	V	0	0	0	0	0	3	191	0,30	0,23
**20**	DFNA-9	56	V	0	0	0	0	0	3	6	0,13	0,11
**21**	Chemotherapy	46	M	0	0	0	0	0	28	154	0,76	0,47
**22**	Familial	64	V	0	0	0	0	0	12	-242	0,25	0,46
**23**	Idiopathic	60	M	0	3	0	0	3	2	98	0,43	0,35

Overview of the characteristics and measurements of the study population.
